# Public Attitudes Toward Ethics and Practices in End-of-Life Decision-Making for Neonates

**DOI:** 10.1001/jamanetworkopen.2023.53264

**Published:** 2024-01-25

**Authors:** Katja Schneider, Stephanie Roll, Tatjana Tissen-Diabaté, Christoph Bührer, Lars Garten

**Affiliations:** 1 Department of Neonatology, Gemeinnützige Gesellschaft der Franziskanerinnen zu Olpe, Marien-Hospital, Bonn, Germany; 2 Department of Neonatology, Charité–Universitätsmedizin Berlin, Berlin, Germany; 3 Institute of Social Medicine, Epidemiology and Health Economics, Charité–Universitätsmedizin Berlin, Berlin, Germany

## Abstract

**Importance:**

Attitudes toward end-of-life decision-making in neonatology have been studied in physicians and other health care professionals and are mostly shaped by their clinical education and work experiences. In contrast, attitudes among the general public have not yet been investigated.

**Objective:**

To assess (1) attitudes in the general public toward euthanasia and withdrawal of life-prolonging treatment in neonates with severe life-limiting conditions, (2) knowledge of current German recommendations, and (3) values in the German society regarding ethical issues and proxy decisions at the beginning of life.

**Design, Setting, and Participants:**

This cross-sectional study was performed in Germany and used an exploratory design to analyze responses to an interview conducted by an independent, established commissioned polling institute in March and April 2022. Participants were 16 years or older, with German language fluency and comprehension and living in Germany.

**Main Outcomes and Measures:**

Knowledge about recommendations for euthanasia and withdrawal of life-prolonging treatment as well as personal attitudes toward (1) euthanasia and withdrawal of life-prolonging treatment and (2) surrogate end-of-life decision-making for newborn infants were assessed.

**Results:**

The study included 2116 participants (1077 females [50.9%]; mean [SD] age 52.1 [18.7] years). Of the participants, 16.8% (311 of 1851) reported knowing the German recommendations for euthanasia and withdrawal of life-prolonging treatment for neonates. Euthanasia and withdrawal of life-prolonging treatment were supported by 64.7% (1369 of 2116) and 77.9% (1649 of 2116) of respondents, respectively. Shared decision-making between parents and physicians for neonates in end-of-life situations was supported by 65.6% of participants (1388). In situations where shared decision-making was not possible, 73.4% of respondents (1019 of 1388) put the ultimate decision to the parents. The magnitude of the associations was low between sociodemographic factors and views on ethical issues and customary practices involved in end-of-life decisions for neonates.

**Conclusions and Relevance:**

Results of this cross-sectional study suggested that most respondents were not aware of the national German recommendations for euthanasia and withdrawal of life-prolonging treatment for sick and extremely preterm newborns. When counseling parents of periviable newborns, clinicians may need to exert more effort in explaining the legal and ethical framework; a highly individualized approach is warranted.

## Key Points

**Question:**

Does the German public know about national recommendations for euthanasia and withdrawal of life-prolonging treatment for neonates, and what personal attitudes exist in German society regarding ethical issues and proxy decisions for newborns with severe life-limiting conditions?

**Findings:**

In this cross-sectional study of 2116 participants, only 17% of participants reported knowing the recommendations regarding euthanasia and withdrawal of life-prolonging treatment for neonates. Associations between sociodemographic factors and views on ethical issues concerning neonatal end-of-life decisions were low in magnitude.

**Meaning:**

Findings of this study suggest that clinicians need to exert additional efforts to explain the legal and ethical framework of end-of-life decisions when counseling parents of periviable infants and that the large variability in attitudes warrants a highly individualized approach.

## Teaser

This cross-sectional study examines the societal perspective on euthanasia and withdrawal of life-prolonging treatment for sick and extremely preterm newborns in Germany.

## Introduction

Should all life-sustaining treatment options be exhausted for every newborn? This question arises often when chances of survival for sick or extremely preterm infants are slim and when permanent health impairments or disabilities are likely. Studies addressing proxy end-of-life decisions in neonatology have largely dealt with questions regarding value concepts and decision-making responsibility, using questionnaires or personal interviews primarily of medical professionals.^1,2,3,4,5,6,7,8^ There are comparative surveys from different clinicians (physicians and nurses) and disciplines (ethics, law, and medicine) and conducted in a number of countries that reveal a country-specific, cultural approach to the professional handling of ethical issues in neonates.^9,10,11,12,13,14,15,16,17,18^

Since 1996, almost all European countries have implemented specific guidelines for addressing ethical aspects of euthanasia, redirection of care, withdrawal of life-prolonging treatment, and medical decision-making for neonates with life-limiting conditions. The requirement of parental involvement is common to all of these guidelines. Most guidelines, including the German one, consider withdrawal of life-prolonging treatment but not euthanasia to be an acceptable option for medical decisions. Two surveys, 1 conducted in 15 French neonatal intensive care units^19^ and the other conducted in 170 Austrian, Swiss, and German^20^ neonatal intensive care units, found that parental involvement in decision-making for neonates with life-limiting conditions has gained increased acceptance among neonatologists. However, there are no data on the views of nonclinicians. Parents of neonates with life-limiting conditions share the inescapable burden of having to make momentous therapeutic decisions at the beginning of their child’s life that will carry lifetime consequences. Any resulting permanent health impairment or disability of the newborn will have long-lasting implications for the parents’ life. Medical professionals, on the other hand, may have an array of hidden conflicts of interests. Bridging the gap between parents and clinicians requires exploring and acknowledging potential differences in values regarding end-of-life decisions.

The primary aim of this study was to assess (1) attitudes in the general public toward euthanasia and withdrawal of life-prolonging treatment in neonates with severe life-limiting conditions, (2) knowledge of current German recommendations, and (3) values in the German society regarding ethical issues and proxy decisions at the beginning of life. Additionally, the associations between values and sociodemographic factors were explored.

## Methods

### Design and Participants

For this cross-sectional study, a population-based, nationwide, representative sample in Germany was recruited and selected for an interview by an independent, established polling institute, the Institut für Demoskopie Allensbach (IfD Allensbach). The Ethikkommission der Charité approved the study. Informed consent was not required from participants under data protection law, as the data were processed, evaluated, and passed on in anonymized form (eMethods in Supplement 1). We followed the Strengthening the Reporting of Observational Studies in Epidemiology (STROBE) reporting guideline.^21^

Inclusion criteria for participants were as follows: (1) age 16 years or older (given that aspects of neonatal end-of-life decisions can affect all citizens of reproductive age, the age limit was set at 16 years; due to the complexity and seriousness of the issue, a lower age limit was not set); (2) sufficient German language fluency and comprehension (participants had to be able to follow the interview in German and answer all questions); and (3) registered residence in Germany.

### Data Collection and Questionnaire Variables

The IfD Allensbach selected study participants by quota sampling with the aim of obtaining a representative sample of the German population. To reduce selection bias, the sample was compiled based on the characteristics of the most recent German microcensus (Microcensus 2020). The interviews were conducted in German in March and April 2022. Detailed information on recruitment and data collection procedure by IfD Allensbach is provided in the eMethods in Supplement 1.

The interview questionnaire (eAppendix in Supplement 1) consisted of questions from the Ethical Decision Making in Neonatal Intensive Care Units Survey (EURONICS), which was led by Cuttini and colleagues,^14,15,16^ that were adapted for better understanding by laypersons. For example, we substituted *withdrawal of life-prolonging treatment* with *passive euthanasia*, the commonly used term in Germany, making the concept more familiar to the general public. In addition, self-designed questions on knowledge of German recommendations addressing euthanasia, withdrawal of life-prolonging treatment, and medical decision-making for neonates were included.

Participants were considered to be personally involved in end-of-life decisions if at least 1 of the following 3 conditions was present: (1) involvement through personal prematurity or physical or mental disability, (2) involvement through own child’s or children’s prematurity or physical or mental disability, or (3) involvement through being a representative for others or medical surrogate (eg, decision-making on withdrawal of life-prolonging treatment or emergency treatment). Participants with such involvement were analyzed as a subgroup.

Overall, we assessed participants’ knowledge and attitudes about euthanasia and withdrawal of life-prolonging treatment, attitudes about end-of-life decision-making responsibility for neonates, and values or ethical attitudes regarding limits to viability based on 13 statements. Sociodemographic variables collected included age, sex, educational level, employment status, household size, children age, marital status and living situation, religious affiliation, and political affiliation.

### Sample Size Determination 

The study had an exploratory design. It had no primary end point (or primary hypothesis) nor any adjustments to control the overall type I error rate. With a sample size of 2000, sufficient precision (approximately 3% expressed by a 95% CI) for a prevalence of a binary outcome of 50% could be achieved with a 99% probability or greater.

### Statistical Analysis

The characteristics of study participants were analyzed using descriptive methods, including absolute and relative frequencies and 95% CIs. Univariable logistic regression models were used to determine associations of various characteristics with responses regarding euthanasia and withdrawal of life-prolonging treatment and ethical attitudes. In addition, age, sex, and prior involvement were included as covariates, along with other variables identified as potentially relevant by univariable analyses. To conduct binary comparisons, we combined the response categories for the attitude toward euthanasia and withdrawal of life-prolonging treatment, and the disapprove category was compared with the combined approve and undecided categories. Similarly, for the knowledge variables, the aware category was compared with the combined not aware and would have assumed categories. The k-modes clustering method was used to identify subgroups of participants with similar ethical attitudes based on their agreement with ethical statements. To determine the optimal number of clusters, we used the elbow method. The resulting clusters were described and compared based on sociodemographic and other relevant characteristics.

All analyses were considered exploratory, without level of significance or adjusting for multiple comparisons. The data included weights, but no weighting was applied in the study results analysis as it had minimal implications for the results. Missing data were not imputed. The statistical analyses were performed with R, version 4.2.1 (R Project for Statistical Computing).

## Results

A total of 2116 individuals were interviewed from March 9 to April 6, 2022. Participants had a mean (SD) age of 52.1 (18.7) years and included 1077 females (50.9%) and 1039 males (49.1%) (Table 1). A subgroup of 410 participants (19.4%) were personally involved in the past in end-of-life decisions or complications at the beginning of life, with the majority (254 of 410 [62.0%]) belonging to the subcategory representatives for others (eFigure 1 in Supplement 1).

**Table 1.** Sociodemographic Characteristics of Participants

Characteristic	Participants, No. (%)
Total No. of participants^a^	2116
Sex	
Female	1077 (50.9)
Male	1039 (49.1)
Age group, y	
16-19	103 (4.9)
20-29	232 (11.0)
30-39	218 (10.3)
40-49	390 (18.4)
50-59	378 (17.9)
60-69	299 (14.1)
≥70	496 (23.4)
Age, mean (SD), y	52.1 (18.7)
Educational level	
Lower secondary level graduation: Hauptschulabschluss	446 (21.1)
Lower secondary level graduation: Realschulabschlussabschluss	708 (33.5)
Upper secondary level graduation: Fach-/Hochschulreife	962 (45.5)
Employment status	
Working full- or part-time	1219 (57.6)
Not working (eg, unemployed, student)	249 (11.8)
Retired	648 (30.6)
Monthly income, € (n = 1967)	
<2500	650 (33.0)
2500-3999	723 (36.8)
≥4000	594 (30.2)
Household size, No. of persons	
1	531 (25.1)
2	947 (44.8)
3	302 (14.3)
4	265 (12.5)
≥5	71 (3.3)
Marital status	
Single, living alone	424 (20.0)
Unmarried, living as a couple	176 (8.3)
Married	1085 (51.3)
Divorced	239 (11.3)
Widowed	192 (9.1)
Parental status and age of youngest child (n = 2115)	
No children	692 (32.7)
Children aged <6 y	155 (7.3)
Children aged 6-17 y	296 (14.0)
Children aged ≥18 y	972 (46.0)
Religious affiliation (n = 2113)	
None	927 (43.9)
Protestant	619 (29.3)
Catholic	496 (23.5)
Muslim	24 (1.1)
Other^b^	47 (2.2)
Political affiliation (n = 2064)	
Christian-democratic (center right)	553 (26.8)
Social-democratic (center left)	628 (30.4)
Economic liberalism	196 (9.5)
Green	397 (19.2)
Democratic socialist (far left)	153 (7.4)
Conservative populist (far right)	137 (6.6)
Residence	
Village (<5000 inhabitants)	292 (13.8)
Small town (5000-20 000 inhabitants)	481 (22.7)
Medium-sized town (>20 000-100 000 inhabitants)	650 (30.7)
Large city (>100 000 inhabitants)	693 (2.8)

^a^ Number of respondents was different for some variables due to missing responses.

^b^ Other was one of the response options; no specific information was available.

### Knowledge and Attitudes About Euthanasia and Withdrawal of Life-Prolonging Treatment

**Figure 1.**
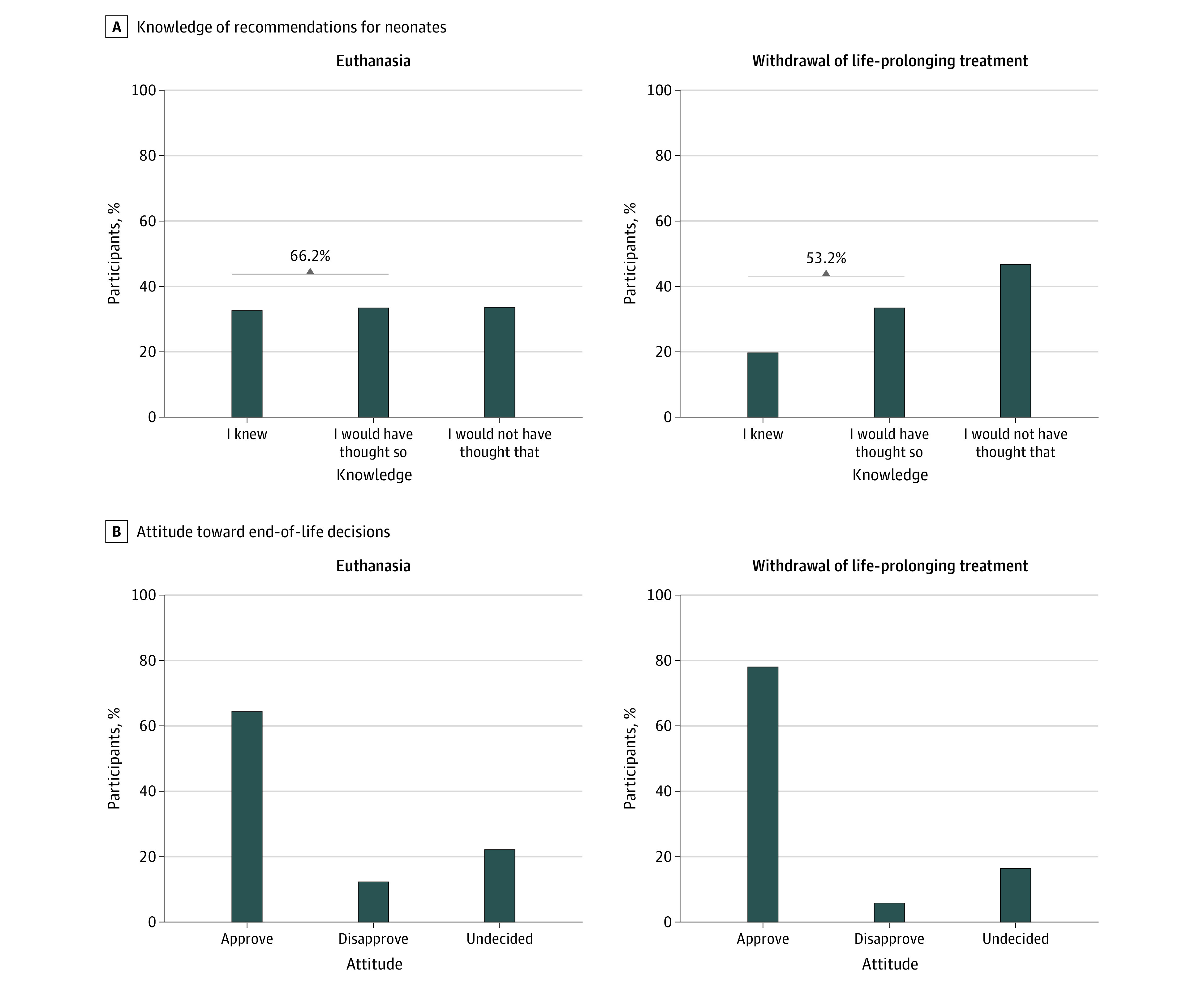
Knowledge of Recommendations for Neonates for Euthanasia and Withdrawal of Life-Prolonging Treatment and General Attitudes Toward End-of-Life Decisions

While 32.7% of participants (630 of 1926) were aware that euthanasia is not legally permitted for neonates in Germany, 19.9% (380 of 1905) were aware that withdrawal of life-prolonging treatment is legally permissible (Figure 1; eTable 1 in Supplement 1). Only 16.8% of respondents (311 of 1851) reported knowledge of both German recommendations for neonatal euthanasia and withdrawal of life-prolonging treatment. Associations were found between higher awareness of euthanasia prohibition and several characteristics, including female sex (vs male sex: 35.1% [347] vs 30.2% [283]; odds ratio [OR], 0.80 [95% CI, 0.66-0.97]), personal involvement (vs without such involvement: 40.1% [154] vs 30.9% [476]; OR, 1.50 [95% CI, 1.19-1.89]), higher educational levels (vs lower levels: 35.6% [315] vs 32.1% [209] vs 27.2% [106]; OR for higher vs lower, 1.48 [95% CI, 1.14-1.93]), and church membership (vs non–church membership, 35.3% [382] vs 29.0% [241]; OR, 1.33 [95% CI, 1.10-1.62]) (eTable 2 in Supplement 1).

The use of withdrawal of life-prolonging treatment currently permitted in Germany was viewed favorably by 77.9% of respondents (1649 of 2116). Despite being explicitly prohibited by law in Germany, 64.7% of respondents (1369 of 2116) favored the use of euthanasia (Figure 1; eTable 1 in Supplement 1). Among participants, 56.5% (1195 of 2116) stated that they saw no difference between discontinued life-prolonging measures and euthanasia for a person with a life-limiting illness. Church members (1189 of 2100) were noticeably more likely to disapprove of euthanasia than non–church members (16.7% [198] vs 7.5% [68]; OR, 2.48 [95% CI, 1.86-3.33]) and were more disapproving of withdrawal of life-prolonging treatment (6.6% [78] vs 4.5% [41]; OR, 1.49 [95% CI, 1.02-2.12]) (eTable 2 in Supplement 1).

### Attitudes About End-of-Life Decision-Making for Neonates

Regarding the question, “When it comes to whether life-prolonging treatment should be withdrawn in newborns who have very little chance of survival or whether intensive medical treatment should be continued: How do you think this is regulated in Germany, who ultimately decides this? The attending physicians, or the parents, or both together?” participants responded as follows: physicians and parents together (39.9% [845]), parents alone (11.8% [250]), physicians alone (11.3% [239]), and undecided or do not know (37.0% [782]).

**Figure 2.**
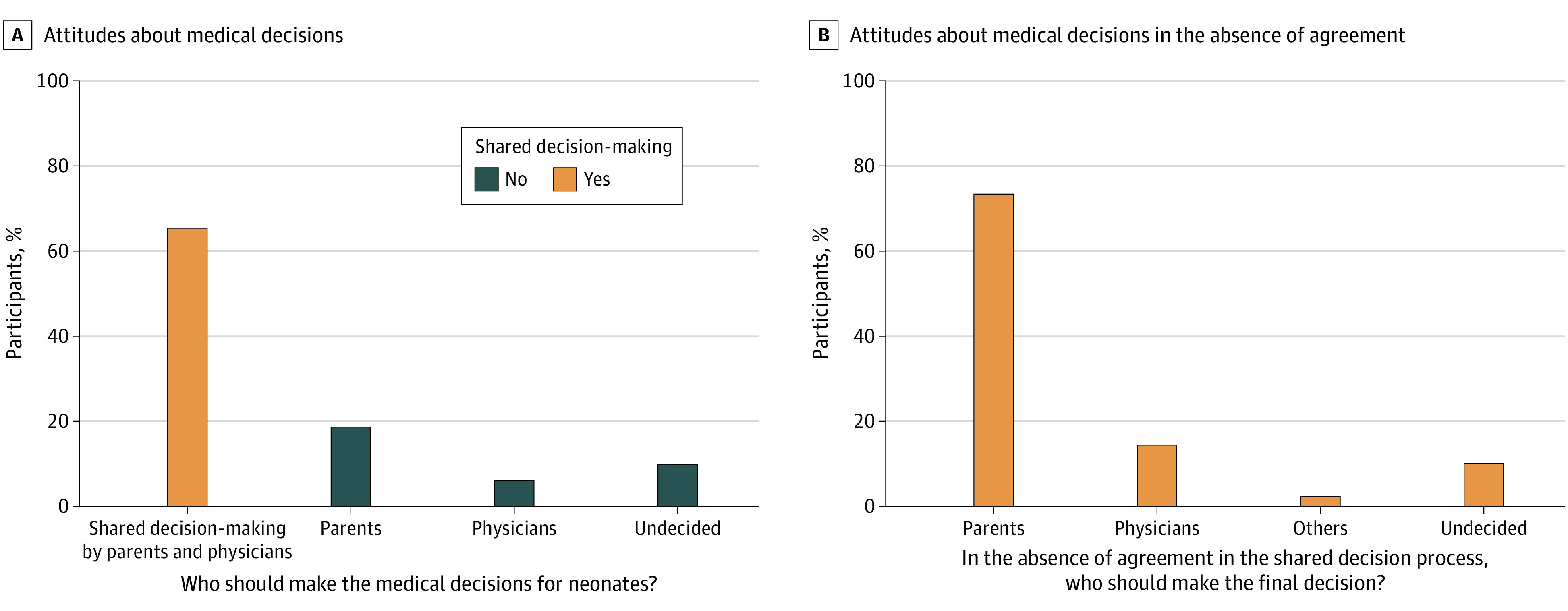
Attitudes About the Primary Decision Maker for Neonatal End-of-Life Decision-Making

When asked who, in their opinion, should make decisions regarding withdrawal of life-prolonging treatment for newborns regardless of current recommendations, most participants (65.6% [1388]) stated that parents and physicians should share the decision-making (Figure 2; eTable 1 in Supplement 1). Among the subgroup of participants with proper knowledge of German recommendations, this shared decision-making response appeared more frequently (74.8% [632 of 845]).

In a situation where no agreement could be reached within the framework of a shared decision-making process, 73.4% of respondents (1019 of 1388) favored the parents making the final decision. Within this group the question, “How much should parents factor the advice and opinions of doctors into their decision?” received a strong or very strong response from 70.6% of participants (278 of 394).

Of all participants, 18.6% (394 of 2116) stated that decisions regarding withdrawal of life-prolonging treatment for newborns should be made by the parents alone. Only 6.1% of respondents (129 of 2116) stated that such decisions should be made solely by physicians. Within this group of respondents, 59.7% (77 of 129) gave either very strong or strong support to the statement, “Doctors should consider the parents’ wishes and opinion in their decision,” while 10.1% (13 of 129) described their support as hardly or not at all and 8.5% (11 of 129) were undecided.

Participants who preferred physicians over parents as the sole decision-maker for end-of-life decisions in newborns were given a list of potential rationales. Among the 129 respondents in this group, 122 (94.6%) agreed with at least 1 of the reasons (multiple responses possible). The argument that only physicians have the medical expertise to determine the best solution received the most support (86.8% [112]). An overview of the arguments supporting physician-made decisions and the rates of agreement is shown in Table 2.

**Table 2.** Participant Agreement With Statements Supporting Physicians as the Sole Decision-Maker for Neonates Regarding Withdrawal of Life-Prolonging Treatment

Statement^a^	I agree response, No. (%)
Only physicians have the medical expertise to determine the best solution.	112 (86.8)
Physicians are more likely to make reasonable and prudent decisions in this situation than parents, who are much more emotionally involved.	93 (72.1)
Parents are often not in the right mental state to make these kinds of decisions.	90 (69.8)
Physicians have the experience to deal with the pressures involved in making such decisions.	71 (55.0)
There is a risk that parents will later regret their decision and feel guilty.	63 (48.8)
I would feel overwhelmed as a parent with a decision like this.	61 (47.3)
Parents should be spared the burden of having to make this decision in such a situation.	57 (44.2)

^a^ Participants were permitted to agree with more than 1 statement.

### Values and Quality of Life

The statement, “The most important factor for quality of life is how independently one can live one’s life” found agreement among 70.4% of respondents (1489 of 2116), whereas 49.9% (1056 of 2116) rejected “fundamental and unconditional protection of life by all means.” Among the respondents, 47.1% (996 of 2116) believed that physicians at the time of decision-making do not give enough consideration to the individual’s posttreatment quality of life. Over half (51.7% [1093 of 2116]) stated that, in Germany, the wishes of patients or their surrogates are often not sufficiently considered in the context of end-of-life decisions.

Participants were presented with statements on aspects of the value of life, quality of life, and euthanasia and withdrawal of life-prolonging treatment for newborns and were asked to respond with I agree, I do not agree, or I do not know. The statement, “Not keeping the child alive with all possible means is justifiable if a newborn child would potentially only survive with severe disabilities” had the highest agreement rate (61.8% [1308]), which was in line with the responses related to quality of life (Table 3). Of all respondents, 50.1% (1061) did not agree with the statement, “The family’s dynamics and social situation should play a role in decisions about life-prolonging measures for newborns who have only a small chance of survival.”

**Table 3.** Participants’ Attitudes About Value of Life, Quality of Life, Euthanasia, Withdrawal of Life-Prolonging Treatment, and End-of-Life Decisions for Neonates

Statement	Responses, No. (%)		
	I agree	I do not agree	Undecided
Not keeping the child alive with all possible means is justifiable if a newborn child would potentially only survive with severe disabilities.	1308 (61.8)	537 (25.4)	271 (12.8)
For people who are severely disabled from birth, I sometimes think that it might have been better for them if they had not been kept alive at all costs.	1183 (55.9)	667 (31.5)	266 (12.6)
In deciding about life-prolonging measures for a critically ill newborn the issue of possible severe lifelong physical disability should not play a role.	895 (42.3)	900 (42.5)	321 (15.2)
The family’s dynamics and social situation should play a role in decisions about life-prolonging measures for newborns who have only a small chance of survival.	813 (38.5)	1061 (50.1)	242 (11.4)
Whether life-sustaining measures should be carried out should be more closely scrutinized in seriously ill elderly people than for seriously ill children.	791 (37.4)	1110 (52.4)	215 (10.2)
The question of severe lifelong [intellectual disability] should not play a role in decisions regarding life-prolonging measures in a critically ill newborn.	770 (36.4)	1008 (47.6)	338 (16.0)
Treatment decisions affecting newborns with very low chances of survival should also take into account the long-term costs of life-prolonging therapeutic interventions.	555 (26.2)	1309 (61.9)	252 (11.9)
One should keep every seriously ill child alive as long as possible, in order to gain knowledge for the treatment of future patients.	338 (16.0)	1497 (70.7)	281 (13.3)

To explore the associations between sociodemographic factors and participant response patterns, we conducted a cluster analysis. The results revealed 3 distinct groups based on their responses to 13 statements concerning ethical considerations and quality of life (eFigure 2 in Supplement 1). Cluster 1 (quality of life; n = 1167) prioritized quality of life and the consideration of potential future disability in treatment decisions (life is not unconditionally worth protecting). Cluster 2 (protection of life; n = 690) strongly opposed the termination of life-prolonging treatment, prioritizing the preservation of life above all else (unconditional protection of life). Cluster 3 (nonpolarized; n = 259) valued independence as the most important quality-of-life aspect and had a nonpolarized attitude toward the protection of life.

Results of the cluster analysis revealed that certain ethical statements were particularly effective in distinguishing between participants with different attitudes. Two statements regarding relevance of future physical disability for treatment decisions and keeping neonates alive by all possible means despite severe disability were sufficient to create distinct groups. Unless explicitly mentioned, no overall associations were found between sociodemographic characteristics and participant responses. In addition, no fundamental differences were found in the answers by respondents in the involvement subgroup compared with the rest of the study population.

## Discussion

To our knowledge, this study was the first representative, population-based, cross-sectional study to address societal values regarding ethical issues at the beginning of life and proxy medical decision-making for newborns with limited viability. Only 16.8% of participants were aware of existing national recommendations in Germany for euthanasia and withdrawal of life-prolonging treatment, and more than one-third did not know (37.0%) who can decide on withdrawal of life-prolonging treatment for a newborn. These findings highlight substantial knowledge gaps, which carry the risk of being filled with myths and misconceptions. What interventions could be implemented to address these knowledge gaps in German society? On a national level, knowledge about the ethical and legal aspects in end-of-life decisions can be delivered through public education campaigns modeled after campaigns with a palliative care context.^22,23,24,25^ On an individual level, neonatologists can explain basic terminology to parents as part of a discussion about end-of-life decision-making and can provide information about legal regulations, national recommendations, and medical ethics. This intervention may proactively dispel parental uncertainties, thereby removing 1 of many recognized barriers perceived by parents when making surrogate end-of-life decisions^26,27^ for their newborns.

Approximately half of respondents (56.5%) stated that it made no difference to them whether life-prolonging measures were discontinued or a lethal drug was administered (euthanasia). In line with this response, almost two-thirds of respondents (64.7%) supported the use of euthanasia in Germany. This level of popular support corresponds to previous reports from western Europe^28,29^ and North America,^30,31,32^ which have found since the 1990s a relatively stable, culture-specific approval rate for euthanasia in approximately two-thirds of the population.^33,34^

Shared decision-making and, in the absence of consensus, final decision-making by parents were supported by the majority of participants (65.6%). This majority opinion aligns with existing national and international guidelines and recommendations regarding shared decision-making.^35,36,37,38,39,40^

Having “medical expertise” was a frequently cited reason for physicians being the sole decision-maker (86.8%). Previous studies have similarly identified parental perception of this asymmetry in medical knowledge as a recurring and substantial barrier to their participation in shared end-of-life decision-making.^26,27,41,42^

Quality of life has evolved as pivotal for medical decision-making in neonatology.^43^ However, there is little consensus on its definition and objective measurements.^44,45,46^ For 70.4% of respondents, the most important factor in assessing quality of life was how independently one can live alone. Respondents drew no relevant distinction between possible physical or mental disability when making end-of-life decisions. Physicians should come forward to address expected independence in later life during discussions with parents regarding neonatal end-of-life decisions.

Only half of the respondents (50.1%) stated that the family dynamics and the social situation of the family should affect end-of-life decisions for newborns. In contrast, socioeconomic and other family factors have been repeatedly demonstrated to heavily alter clinicians’ medical decisions for neonates.^47,48^

Almost half of the respondents in this study (47.1% and 51.7%) believed that physicians do not give enough consideration to the individual’s posttreatment quality of life when making important treatment decisions and that patients or surrogates are insufficiently integrated into end-of-life decision-making. The ongoing gap between guidelines and reality^36,49,50,51^ is a reflection of findings in a previous survey of German-speaking neonatologists,^20^ almost 20% of whom admitted considering resuscitating extremely preterm infants against parental wishes.

Study participants with prior personal involvement did not reveal attitudes that were fundamentally different from those expressed by the general public. Furthermore, we were surprised by the low-magnitude associations between sociodemographic variables and attitudes toward euthanasia and withdrawal of life-prolonging treatment, in contrast to findings in other studies that have shown associations with variables such as age, educational level, sex, income level, religiosity, or health-related factors with certain attitudes toward euthanasia, withdrawal of life-prolonging treatment, and end-of-life decision-making.^52,53,54,55,56^ We assumed that, in a culturally diverse society, personal views become less determined by sociodemographic variables than in the past.

### Limitations

The scope of this study was limited by the cross-sectional design, which does not allow for causal inferences, and the exploratory approach. Restricting participants to those with sufficient German language skills implies omission of a large fraction of recent immigrants. Given that the study sample was compiled with the aim of reflecting the representative characteristics of the German microcensus, the proportion of participants most likely to make proxy decisions for newborns (adults aged 20-45 years) was limited. A lack of personal exposure might have affected the results regarding newborn-specific questions.

## Conclusions

In this cross-sectional study of public attitudes toward end-of-life decision-making for neonates, most respondents were not familiar with German recommendations for euthanasia and withdrawal of life-prolonging treatment for sick and extremely preterm newborns. In contrast to legally and ethically upheld positions, most respondents made no distinction between euthanasia and withdrawal of life-prolonging treatment, regarding euthanasia as a permissible option. The magnitude of the association was low between sociodemographic variables and views on euthanasia and withdrawal of life-prolonging treatment. Physicians counseling parents of periviable newborns should exert more effort into explaining the legal and ethical framework of end-of-life decisions, be aware that shared decision-making was favored by most participants, and consider a highly individualized approach owing to the large variability in attitudes.

## Notes

Supplement 1. **eMethods.** Detailed Information on Recruitment, Data Collection Procedure and Ethical Considerations **eAppendix.** Questionnaire as Used by the Polling Institute IFD Allensbach (Original in German, Given Here a Simple English Translation) **eFigure 1.** Subgroup Analysis of 410 Respondents Who Were Involved in the Past in Medical Decisions or Complications at the Beginning of Life; Descriptive Analysis **eTable 1.** Knowledge of Euthanasia and Withdrawal of Life-Prolonging Treatment Regulations and End-of-Life Decision Making in Neonates **eTable 2.** Associations Between Socio-Demographic Factors and Respondents' Attitudes Towards Euthanasia and Withdrawal of Life-Prolonging Treatment, As Well As Their Knowledge of the Respective Regulations (Results From Unadjusted and Adjusted Logistic Regression; Adjustment for All Variables Listed) **eFigure 2.** Frequency of Agreement and Disagreement to the 13 Statements Grouped by Cluster

Supplement 2.
Data Sharing Statement
