# Supplementary material for: Public Attitudes Toward Ethics and Practices in End-of-Life Decision-Making for Neonates
Source: JAMA Netw Open. 2024 Jan 25;7(1):e2353264. doi: 10.1001/jamanetworkopen.2023.53264 (PMC10811557; doi:10.1001/jamanetworkopen.2023.53264)
Supplement: Supplement 1. — eMethods. Detailed Information on Recruitment, Data Collection Procedure and Ethical Considerations eAppendix. Questionnaire as Used by the Polling Institute IFD Allensbach (Original in German, Given Here a Simple English Translation) eFigure 1. Subgroup Analysis of 410 Respondents Who Were Involved in the Past in Medical Decisions or Complications at the Beginning of Life; Descriptive Analysis eTable 1. Knowledge of Euthanasia and Withdrawal of Life-Prolonging Treatment Regulations and End-of-Life Decision Making in Neonates eTable 2. Associations Between Socio-Demographic Factors and Respondents' Attitudes Towards Euthanasia and Withdrawal of Life-Prolonging Treatment, As Well As Their Knowledge of the Respective Regulations (Results From Unadjusted and Adjusted Logistic Regression; Adjustment for All Variables Listed) eFigure 2. Frequency of Agreement and Disagreement to the 13 Statements Grouped by Cluster [file jamanetwopen-e2353264-s001.pdf]

Supplemental Online Content

Schneider K, Roll S, Tissen-Diabaté T, Bührer C, Garten L. Public attitudes toward ethics and practices in end-of-life decision-making for neonates. *JAMA Netw Open*. 2024;7(1):e2353264. doi:10.1001/jamanetworkopen.2023.53264

eMethods. Detailed Information on Recruitment, Data Collection Procedure and Ethical Considerations

eAppendix. Questionnaire as Used by the Polling Institute IFD Allensbach (Original in German, Given Here a Simple English Translation)

eFigure 1. Subgroup Analysis of 410 Respondents Who Were Involved in the Past in Medical Decisions or Complications at the Beginning of Life; Descriptive Analysis

eTable 1. Knowledge of Euthanasia and Withdrawal of Life-Prolonging Treatment Regulations and End-of-Life Decision Making in Neonates

eTable 2. Associations Between Socio-Demographic Factors and Respondents' Attitudes Towards Euthanasia and Withdrawal of Life-Prolonging Treatment, As Well As Their Knowledge of the Respective Regulations (Results From Unadjusted and Adjusted Logistic Regression; Adjustment for All Variables Listed)

eFigure 2. Frequency of Agreement and Disagreement to the 13 Statements Grouped by Cluster

This supplemental material has been provided by the authors to give readers additional information about their work.

© 2024 Schneider K et al. *JAMA Network Open*

eMethods. Detailed Information on Recruitment, Data Collection Procedure and Ethical Considerations

For this study an external, independent, and established German polling institution - the ‘Institut für Demoskopie Allensbach’ (IfD Allensbach) - was commissioned. IfD Allensbach has around 90 permanent employees. With around 1,200 interviewers, it has one of the largest field organizations for face-to-face interviews in Germany.

Recruitment and training of interviewers

When interviewers were recruited, training documents were provided and trial interviews had to be conducted. An accompanying letter was also attached to each survey, in which further information were provided on the survey methodology and special features of the survey. If the interviewers had any queries, they could contact the relevant department at the institute by telephone. In the event of incorrectly completed questionnaires, feedback was provided to the interviewers.

Recruitment of participants

The IfD Allensbach, an established German polling institution, selected study participants by quota sampling with the aim to obtain a representative sample for the German population.

The sample was compiled based on the characteristics of the German microcensus (Federal Statistical Office, Microcensus 2020). The polling institute has a fixed panel of citizens available for interviewing. It was not done by probabilistic random sampling. Thus, no participant declined study participation as every participant was prepared taking part in the interview.

The regional distribution of the sample is controlled via a stratified deployment according to five municipality size classes in 46 administrative units (federal states/regional districts/county groups). In order to achieve the representative sample, interviewers were given explicit instructions regarding the required number of individuals with certain characteristics (German region, sex, age

groups, employment status, and professional group), thus ensuring that predetermined quotas were met.

Ethical considerations

Prior to the interview, information about the questionnaire were provided to the participants. A further declaration of consent was not required under data protection law, as the data was only processed, evaluated and passed on in a completely anonymized form. To protect confidentiality, no personal data were generated or collected that could be used to link a respondent to her or his response or to distinguish respondents from non-respondents. The selection of participants as well as conduct of the interviews was performed by an external German polling institution (Institut für Demoskopie Allensbach, (IfD Allensbach) with participants being part of a general panel. Thus, there was no direct contact between the clinical researchers and the participants.

© 2024 Schneider K et al. *JAMA Network Open*

eAppendix. Questionnaire as Used by the Polling Institute IFD Allensbach
(Original in German, Given Here a Simple English Translation)

Proxy decisions at the beginning of life

Extract from the main surveys 12051 and 12052 March/April 2022.

INTERVIEWER: Read questions aloud verbatim. Please circle the letters or numbers next to applicable answers. If no answers are given, write answers verbatim on the dotted lines. All results from this survey are used to explore and better communicate the public's opinion.

After some introductory questions on other topics:

1. ""Now to a completely different topic, namely the topic of euthanasia: There is always a discussion about euthanasia. Euthanasia means ending the lives of seriously ill people who are suffering severely or who no longer have a chance of survival, at their own request, for example by giving them a lethal drug. Are you for or against euthanasia?"

1. FOR
2. AGAINST
3. UNDECIDED

---

2. "Euthanasia is not permitted in Germany. This also applies to newborns who are born prematurely or seriously ill or are born seriously ill and have little or no chance of survival. Were you aware that euthanasia is not permitted for such newborns either, or were you not aware of this but you would have suspected it, or would you not have thought that?"

1. I KNEW
2. I WOULD HAVE THOUGHT SO
3. I WOULD NOT HAVE THOUGHT THAT
4. NO INDICATION

---

3. "In addition to euthanasia, withdrawal of life-prolonging treatment is also sometimes discussed. Withdrawal of life prolonging treatment means that a doctor stops or does not even start life-prolonging treatment on a terminally ill patient if the patient or the relatives have expressly stated that this is what the patient wants. Are you in favor of or against withdrawal of life-prolonging treatment?"

4. APPROVE
5. DISAPPROVE
6. UNDECIDED

© 2024 Schneider K et al. *JAMA Network Open*

4. "Withdrawal of life-prolonging treatment is generally permitted in Germany. This also applies to newborns who are born much too early or seriously ill, if the children have no or only very low chances of survival or if continued life would only be possible with severe permanent suffering. Were you aware that withdrawal of life-prolonging treatment is permitted in newborns, or were you not aware of this but you would have suspected it, or would you not have thought so?"

1. I KNEW
2. I WOULD HAVE THOUGHT SO
3. I WOULD NOT HAVE THOUGHT THAT
4. NO INDICATION

---

5. "In your opinion, who should decide whether withdrawal of life-prolonging treatment should be performed or life-prolonging measures should be continued in the case of a newborn baby who has very little chance of survival: the attending physicians or the child's parents?"

1. PHYSICIANS
2. PARENTS
3. PHYSICIANS AND PARENTS TOGETHER
4. UNDECIDED

* „And if physicians and parents do not agree on whether life-prolonging treatment should be withdrawn or continued: Who should have the final say and make the decision in this case?"

1. PHYSICIANS
2. PARENTS
3. DIFFERENT ANSWER, namely: ………………………………………………………………………………
4. UNDECIDED

---

6. INTERVIEWER rating according to questions 5 or 5*:
According to the respondent, who should make the decision to?

PHYSICIANS …………………………… 1*
PARENTS ………………………….. 2***
DIFFERENT ANSWER ………. 3
UNDECIDED …………. 4

© 2024 Schneider K et al. *JAMA Network Open*

*
a) INTERVIEWER presents **pink list 1** !

„You did indicate that you think physicians should make that decision. After this list, can you tell me why you think doctors should make this decision?“

(Circle everything mentioned!)
/ 1 / 2 / 3 / 4 / 5 / 6 / 7
NO INDICATION ………….9

b) „How much should doctors take the parents' wishes into account when making a decision? Would you say …“

"very strong" 1
"strong" 2
"less strong" 3
"hardly, not at all" 4
UNDECIDED 5

***
A) „How much should parents factor the advice and opinions of doctors into their decision? Would you say …“

"very strong" 1
"strong" 2
"less strong" 3
"hardly, not at all" 4
UNDECIDED 5

---

7. "When it comes to whether life-prolonging treatment should be withdrawn in newborns who have very little chance of survival or whether intensive medical treatment should continued: How do you think this is regulated in Germany, who ultimately decides this? The attending physicians, or the parents, or both together?"

1. PHYSICIANS
2. PARENTS
3. PHYSICIANS AND PARENTS TOGETHER
4. UNDECIDED, DON'T KNOW

© 2024 Schneider K et al. *JAMA Network Open*

8. INTERVIEWER hands over **green deck of cards** and **white picture sheet 2**!

"Here on the cards are various statements. Which of these statements would you agree with, and which statements would you disagree with? Please distribute the cards on the sheet accordingly. Please put aside cards for which you cannot make up your mind."
(Circle where applicable!)

AGREE: / 1 / 2 / 3 / 4 / 5 / 6 / 7 / 8 / 9 / 10 / 11 / 12 / 13
DISAGREE: / 1 / 2 / 3 / 4 / 5 / 6 / 7 / 8 / 9 / 10 / 11 / 12 / 13

---

9. INTERVIEWER presents **blue list 3**!

" Do one or more of the items from this list apply to you, or this is not the case?"
(Circle everything mentioned!)

YES, namely: / 1 / 2 / 3 / 4 / 5 / 6 / 7 / 8 / 9 / 10 /
NO, NOT THE CASE X

© 2024 Schneider K et al. *JAMA Network Open*

To question 6 * a)

L I S T 1

(1) Only physicians have the medical
expertise to assess what is
is the best solution

(2) Parents are often not in the mental
condition to make this decision

(3) Parents should be spared the burden,
to make this decision in such a situation.

(4) There is a risk that parents may later regret their
their decision and feel guilty

(5) Physicians are more likely to make
reasonable and prudent decisions in this situation
than parents, who are much more emotionally
are

(6) I would feel overwhelmed as a parent with such a
decision

(7) Physicians have experience with the pressures that
such decisions bring with them

© 2024 Schneider K et al. *JAMA Network Open*

To question 8

WHITE PICTURE SHEET 2

To this statement I would –

AGREE:

DISAGREE:

© 2024 Schneider K et al. *JAMA Network Open*

To question 8

green deck of cards
The following three sheets were cut into individual cards,
on each of which only one of the statements could be read.
The interviewers had to give the respondents
cards into the hands in random order.

1. Not keeping a newborn child alive with all possible means is justifiable if the child would potentially only survive with severe disabilities

2. Life should always be protected unconditionally with all available means available

3. In deciding about life-prolonging measures for a critically ill newborn the issue of possible severe lifelong *physical disability* should NOT play a role

4. The question of severe lifelong *mental retardation* should NOT play a role in decisions regarding life-prolonging measures in a critically ill newborn

© 2024 Schneider K et al. *JAMA Network Open*

5. The family's dynamics and social situation should play a role in decisions about life-prolonging measures for newborns who have only a small chance of survival

6. One should keep seriously ill child alive as long as possible, in order to gain knowledge for the treatment of future patients

7. Treatment decisions affecting newborns with very low chances of survival should also take into account the long-term costs of life-prolonging therapeutic interventions

8. Patients' and surrogates' wishes are insufficiently regarded during medical decisions making

9. Physicians give not enough consideration to the individual's pot-treatment quality of life

© 2024 Schneider K et al. *JAMA Network Open*

10. The most important factor for quality of life is how independently one can live one's life

11. Whether life-sustaining measures should be carried out should be more closely scrutinized in seriously ill elderly people than for seriously ill children

12. There is a great difference between discontinuing life-prolonging measures and administering a lethal drug for critically ill patients

13. For people who are severely disabled from birth, I sometimes think that it might have been better for them if they had not been kept alive at all costs

© 2024 Schneider K et al. *JAMA Network Open*

To question 9

LIST 3

(1) I know of cases in my family or my circle of friends who have had complications during pregnancy or the birth

(2) I know of cases in my family or my circle of friends where a child was born prematurely

(3) I know of cases in my family or circle of friends where a child has a mental or physical disability

(4) My own child had complications during pregnancy or the birth

(5) My own child was born prematurely

(6) My own child has a mental or physical Disability

(7) I myself was born prematurely

(8) I myself live with a mental or physical physical disability

(9) I have already had to make serious medical decisions myself or as a custodian.

(10) I already had to decide as a custodian whether life-sustaining treatment should be continued or withdrawn.

© 2024 Schneider K et al. *JAMA Network Open*

eFigure 1. Subgroup Analysis of 410 Respondents Who Were Involved in the Past in Medical Decisions or Complications at the Beginning of Life; Descriptive Analysis

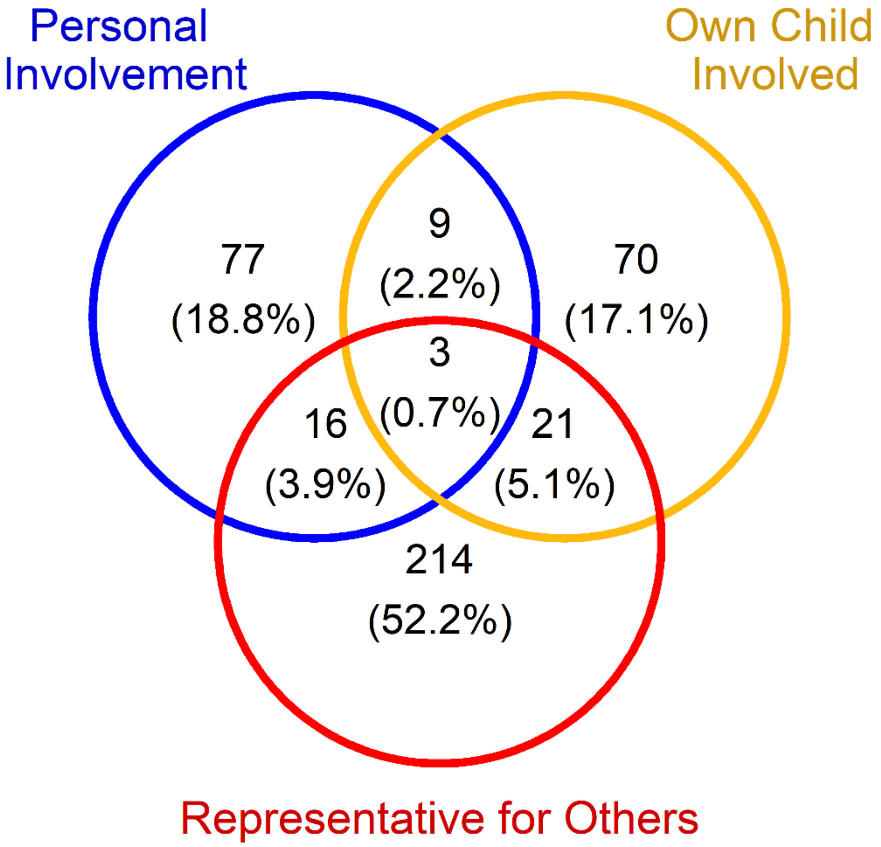

Legend:
Personal involvement: personally affected by prematurity or a physical or mental disability, Own child involved: one or more own children affected by prematurity or physical or mental disability; Representative for others: personal serious medical decision making as a surrogate (e.g., withdrawal of life-prolonging treatment or emergency treatment).

© 2024 Schneider K et al. *JAMA Network Open*

eTable 1. Knowledge of Euthanasia and Withdrawal of Life-Prolonging Treatment Regulations and End-of-Life Decision Making in Neonates

	n (%)
**Knowledge of regulations for neonates**	
**Did you know that euthanasia is not permitted for neonates?**	
I knew	630 (32.7)
I would have thought so	645 (33.5)
I would not have thought that	651 (33.8)
**Did you know that withdrawal of life-prolonging treatment is permitted for neonates?**	
I knew	380 (19.9)
I would have thought so	634 (33.3)
I would not have thought that	891 (46.8)
**Attitude towards end-of-life decisions**	
**Are you in favor of or against active euthanasia?**	
Approve	1,369 (64.7)
Disapprove	269 (12.7)
Undecided	478 (22.6)
**Are you in favor of or against withdrawal of life-prolonging treatment?**	
Approve	1,649 (77.9)
Disapprove	121 (5.7)
Undecided	346 (16.4)
**Primary decision maker for neonatal end-of-life decision making**	
**Who should make the medical decisions for a neonate who has only a very small chance of survival?**	
Shared decision making by parents and physicians	1,388 (65.6)
Parents	394 (18.6)
Physicians	129 (6.1)
Undecided	205 (9.7)
**In the absence of agreement in the shared decision process, who should make the final decision?**	
Parents	1,019 (73.4)
Physicians	201 (14.5)
Others	30 (2.2)
Undecided	138 (9.9)

© 2024 Schneider K et al. *JAMA Network Open*

eTable 2. Associations Between Socio-Demographic Factors and Respondents' Attitudes Towards Euthanasia and Withdrawal of Life-Prolonging Treatment, As Well As Their Knowledge of the Respective Regulations (Results From Unadjusted and Adjusted Logistic Regression; Adjustment for All Variables Listed)

	**Attitude towards euthanasia: „disapprove“** vs. „approve“ or „undecided“		**Attitude towards withdrawal of life-prolonging treatment: „disapprove“** vs. „approve“ or „undecided“		**Knowledge of euthanasia regulation: „aware“** vs. „not aware“ or „would have thought“		**Knowledge of withdrawal of life-prolonging treatment regulation**: **„aware“** vs. „not aware“ or „would have thought“	
	OR (95% CI) (unadjusted)	OR (95% CI) (adjusted)	OR (95% CI) (unadjusted)	OR (95% CI) (adjusted)	OR (95% CI) (unadjusted)	OR (95% CI) (adjusted)	OR (95% CI) (unadjusted)	OR (95% CI) (adjusted)
**Sex male** (reference: female)	0.9 (0.7 – 1.2)	1.0 (0.8 – 1.3)	0.8 (0.6 – 1.2)	0.8 (0.6 – 1.2)	0.8 (0.7 – 1.0)	0.8 (0.7 – 1.0)	0.6 (0.5 – 0.8)	0.6 (0.5 – 0.8)
**Age** (reference: <20)								
20-29	1.4 (0.6 - 3.2)	1.4 (0.7 - 3.4)	0.6 (0.2 - 1.6)	0.5 (0.2 - 1.5)	1.2 (0.7 - 2.2)	1.3 (0.7 - 2.3)	1.5 (0.7 - 3.1)	1.5 (0.7 - 3.1)
30-39	1.4 (0.7 - 3.3)	1.5 (0.7 - 3.5)	0.7 (0.3 - 2.0)	0.7 (0.3 - 1.9)	1.9 (1.1 - 3.3)	1.3 (0.7 - 2.3)	2.2 (1.1 - 4.5)	2.3 (1.2 - 4.8)
40-49	1.4 (0.7 - 3.1)	1.4 (0.7 - 3.3)	0.7 (0.3 - 2.0)	0.8 (0.3 - 2.1)	1.7 (1.0 - 2.9)	1.8 (1.0 - 3.1)	2.2 (1.2 - 4.5)	2.2 (1.2 - 4.5)
50-59	1.4 (0.7 - 3.2)	1.4 (0.7 - 3.2)	0.7 (0.3 - 1.9)	0.8 (0.3 - 2.0)	1.4 (0.9 - 2.5)	1.5 (0.9 - 2.7)	1.4 (0.8 - 2.9)	1.4 (0.8 - 3.0)
60-69	1.6 (0.8 - 3.7)	1.6 (0.8 - 3.6)	1.2 (0.5 - 3.1)	1.2 (0.5 - 3.2)	1.6 (1.0 - 2.9)	1.7 (1.0 - 3.0)	1.2 (0.6 - 2.5)	1.2 (0.6 - 2.5)
≥70	1.9 (1.0 - 4.2)	1.7 (0.9 - 3.9)	0.9 (0.4 - 2.2)	0.9 (0.4 - 2.2)	1.4 (0.9 - 2.4)	1.6 (0.9 - 2.7)	1.4 (0.8 - 2.8)	1.4 (0.7 - 2.9)
**Church membership** (reference: no)	2.5 (1.9 – 3.3)	2.4 (1.8 – 3.3)	1.5 (1.0 – 2.2)	1.4 (1.0 – 2.1)	1.3 (1.1 – 1.6)	1.4 (1.1 – 1.7)	1.5 (1.2 – 1.9)	1.6 (1.2 – 2.0)
**Education** (reference: low):								
**medium**	0.7 (0.5 – 1.0)	0.9 (0.6 – 1.2)	0.8 (0.5 – 1.3)	0.8 (0.5 – 1.4)	1.3 (1.0 – 1.7)	1.3 (1.0 – 1.8)	1.2 (0.9 – 1.7)	1.2 (0.8 – 1.7)
**high**	0.7 (0.5 – 1.0)	0.8 (0.6 – 1.1)	0.7 (0.5 – 1.2)	0.8 (0.5 – 1.3)	1.5 (1.1 – 1.9)	1.6 (1.2 – 2.1)	1.4 (1.1 – 2.0)	1.5 (1.1 – 2.0)

© 2024 Schneider K et al. *JAMA Network Open*

**Personal involvement** (reference: no**)**	1.4 (1.0 – 1.9)	1.4 (1.0 – 1.9)	0.8 (0.5 – 1.3)	0.8 (0.5 – 1.2)	1.5 (1.2 – 1.9)	1.5 (1.2 – 1.9)	1.4 (1.1 – 1.9)	1.4 (1.1 – 1.9)

© 2024 Schneider K et al. *JAMA Network Open*

eFigure 2. Frequency of Agreement and Disagreement to the 13 Statements Grouped by Cluster

Cluster 1 (*'quality of life'*-cluster): prioritized quality of life (QoL) and the consideration of potential future disability in treatment decisions, Cluster 2 (*'sustaining'*-cluster): strongly opposed termination of life-prolonging treatment, prioritizing the preservation of life above all else, and Cluster 3 (*'non-response'*-cluster).

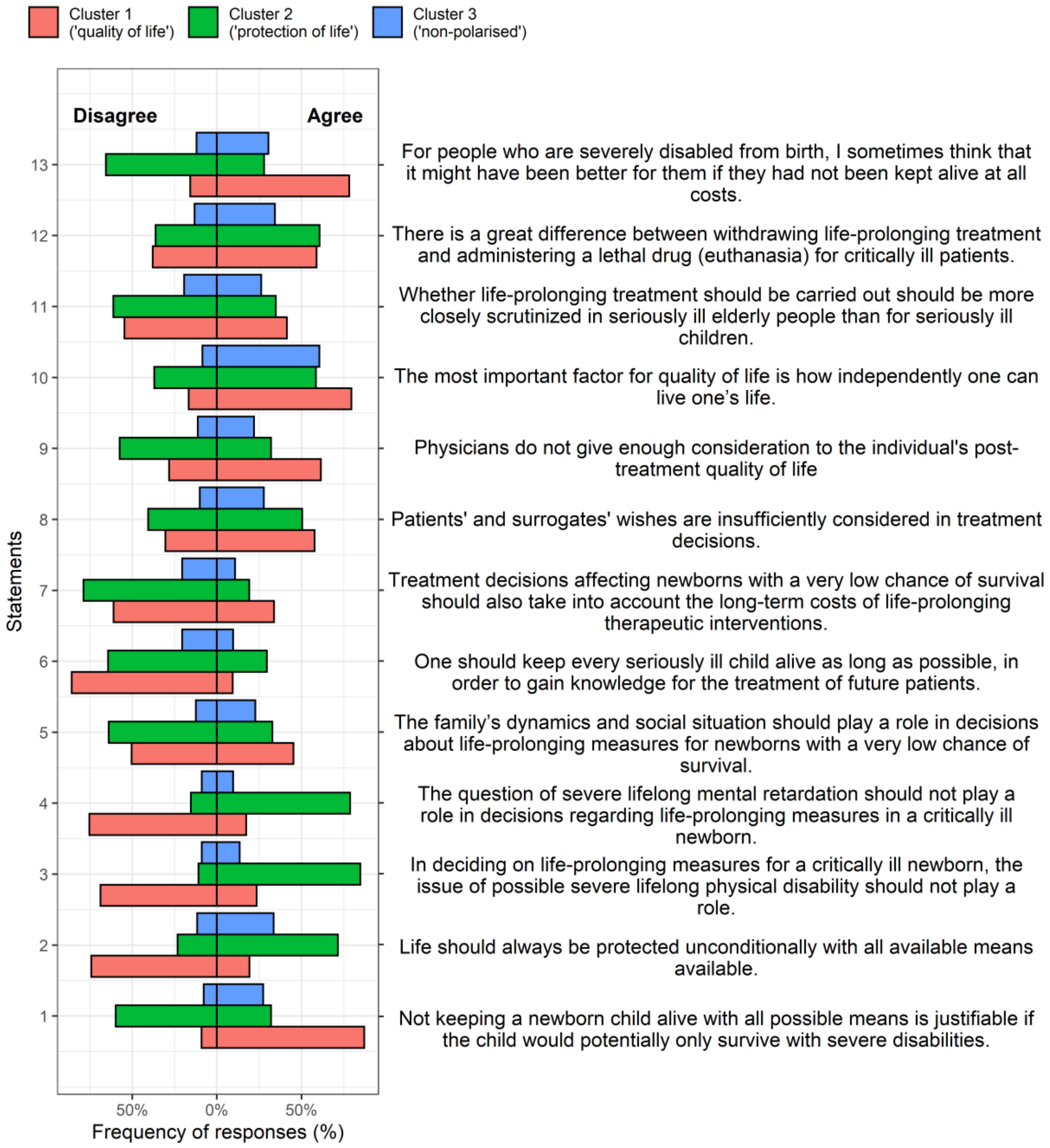

© 2024 Schneider K et al. *JAMA Network Open*
