# Supplementary material for: Public Attitudes Toward Ethics and Practices in End-of-Life Decision-Making for Neonates
Source: JAMA Netw Open. 2024 Jan 25;7(1):e2353264. doi: 10.1001/jamanetworkopen.2023.53264 (PMC10811557; doi:10.1001/jamanetworkopen.2023.53264)
Supplement: Supplement 2. — Data Sharing Statement [file jamanetwopen-e2353264-s002.pdf]

Data Sharing Statement

Schneider. Public Attitudes Toward Ethics and Practices in End-of-Life Decision-Making for Neonates. *JAMA Netw Open*. Published January 25, 2024.
doi:10.1001/jamanetworkopen.2023.53264

Data
Data available: No

Additional Information
Explanation for why data not available: Anonymized data will be provided to researchers with a project approved by an institutional review board after consultation with the data protection officer.
